# Early symptoms preceding post-infectious irritable bowel syndrome following COVID-19: a retrospective observational study incorporating daily gastrointestinal symptoms

**DOI:** 10.1186/s12876-023-02746-y

**Published:** 2023-04-05

**Authors:** Ryo Yamamoto, Asako Yamamoto, Tatsuhiro Masaoka, Koichiro Homma, Tadashi Matsuoka, Ryo Takemura, Michihiko Wada, Junichi Sasaki, Keio Donner Project, Takanori Kanai, Masayuki Amagai, Hideyuki Saya, Hiroshi Nishihara

**Affiliations:** 1 Department of Emergency and Critical Care Medicine, Keio University School of Medicine, Tokyo, Japan; 2 Clinical and Translational Research Center, Keio University Hospital, Tokyo, Japan; 3 Division of Gastroenterology and Hepatology, Department of Internal Medicine, Keio University School of Medicine, 35 Shinanomachi, Shinjuku-Ku, Tokyo, 160-8582 Japan; 4 Department of Gastroenterology and Hepatology, International University of Health and Welfare Mita Hospital, Tokyo, Japan

**Keywords:** Irritable bowel syndrome, COVID-19, Diarrhea

## Abstract

**Background:**

Intestinal microinflammation with immune dysfunction due to severe acute respiratory syndrome coronavirus 2 reportedly precipitates post-infectious irritable bowel syndrome. This study aimed to elucidate potential risk factors for subsequent development of irritable bowel syndrome, hypothesizing that it is associated with specific symptoms or patient backgrounds.

**Methods:**

This single-center retrospective observational study (2020–2021) included adults with confirmed coronavirus disease requiring hospital admission and was conducted using real-world data retrieved from a hospital information system. Patient characteristics and detailed gastrointestinal symptoms were obtained and compared between patients with and without coronavirus disease-induced irritable bowel syndrome. Multivariate logistic models were used to validate the risk of developing irritable bowel syndrome. Moreover, daily gastrointestinal symptoms during hospitalization were examined in patients with irritable bowel syndrome.

**Results:**

Among the 571 eligible patients, 12 (2.1%) were diagnosed with irritable bowel syndrome following coronavirus disease. While nausea and diarrhea during hospitalization, elevated white blood cell count on admission, and intensive care unit admission were associated with the development of irritable bowel syndrome, nausea and diarrhea were identified as risk factors for its development following coronavirus disease, as revealed by the adjusted analyses (odds ratio, 4.00 [1.01–15.84] and 5.64 [1.21–26.31], respectively). Half of the patients with irritable bowel syndrome had both diarrhea and constipation until discharge, and constipation was frequently followed by diarrhea.

**Conclusions:**

While irritable bowel syndrome was rarely diagnosed following coronavirus disease, nausea and diarrhea during hospitalization precede the early signs of irritable bowel syndrome following coronavirus disease.

**Supplementary Information:**

The online version contains supplementary material available at 10.1186/s12876-023-02746-y.

## Background

Coronavirus disease 2019 (COVID-19) mainly affects the lung tissues and causes various respiratory symptoms, which often develop into respiratory failure requiring intensive care [1, 2]. Although significantly high mortality rates of COVID-19 were recorded at the beginning of the pandemic owing to non-standardized care and resource limitations, several medications and vaccinations have prevented disease progression and improved clinical outcomes [3–5]. However, patients are still diagnosed with COVID-19 worldwide without evident subsidence, and some patients who survive COVID-19 develop post-infectious symptoms [6, 7].

Although gastrointestinal (GI) diseases are not common in the early phase of COVID-19 [8, 9], GI symptoms often emerge and persist for several months even in mild-to-moderate COVID-19 [10, 11]. A previous study found that > 20% of patients reported diarrhea and nausea/vomiting during hospitalization and that 5%–10% of patients presented with lingering GI symptoms at 3 months after the initial infection [10]. Considering that the epithelium of the GI tract contains angiotensin-converting enzyme-2 (ACE-2) receptors [12], severe acute respiratory syndrome coronavirus-2 (SARS-CoV-2) enters and affects the digestive tract. Moreover, as several studies have reported that both bacterial and viral infections in the GI tract can cause functional GI disorders (FGID), including irritable bowel syndrome (IBS) [13, 14], long-lasting GI symptoms even after virus clearance would be considered clinical signs of such diseases. A recent study identified that 2%–5% of patients with COVID-19 developed IBS at 6 months after infection [15, 16].

In addition to disruption of the epithelial barrier and intestinal microinflammation due to SARS-CoV-2, systemic immune dysregulation and psychological stress caused by COVID-19 might precipitate post-infectious FGID, particularly IBS [16]. While several studies have attempted to identify conditions before COVID-19 recovery that can predispose patients to post-infectious IBS [15–17], lack of detailed information on daily GI symptoms impedes the validation of underlying pathophysiological mechanisms in post-infectious IBS following COVID-19.

Accordingly, we examined the characteristics and daily GI symptoms of patients with COVID-19 who required hospital admission using detailed electronic data obtained directly from a hospital information system. This study aimed to elucidate the potential risks and preceding early signs of the development of IBS, with the hypothesis that specific symptoms or patient backgrounds would be associated with the development of IBS following COVID-19.

## Methods

### Study design and setting

We conducted a single-center retrospective observational study on patients with COVID-19 confirmed by a positive reverse transcription polymerase chain reaction (RT-PCR) result for SARS-CoV-2. We used data from January 2020 to October 2021, which were obtained directly from the hospital information system of Keio University Hospital, a tertiary care center in Tokyo, Japan. Written informed consent was obtained from all the registry participants. The present study was approved by the Institutional Review Board of Keio University School of Medicine (application number: 20200063).

Several patients with COVID-19 were identified in Japan in early 2020; thereafter, five surges of COVID-19 cases occurred in the study period. During these surges, the governor of Tokyo Metropolis announced several stay-at-home orders, each lasting for 1–2 months [18]. At the study institution, patients with mild-to-moderate COVID-19 who required oxygen, but not mechanical ventilation (MV), were treated by pulmonary internal medicine physicians in general wards, whereas intensive care physicians treated patients with severe COVID-19 who needed MV or extracorporeal membrane oxygenation in the intensive care unit (ICU).

GI symptoms during hospitalization were initially assessed by a treating physician, and gastroenterologists were always consulted when GI symptoms persisted. Although GI diseases can be diagnosed by either a treating physician or gastroenterologist, patients with confirmed or suspected GI diseases are always assessed by a gastroenterologist.

Data were obtained from the Donner Registry, which was established as a real-world data registry at Keio University School of Medicine. The Donner Registry has been prospectively collecting data of patients with COVID-19 from the hospital information system consisting of various records, such as demographics; auto-recorded parameters in patient-monitoring devices; descriptive records by healthcare providers, laboratories, and images; and detailed information on when these data were saved. Diseases diagnosed after admission were also available in the registry with codes using the International Classification of Diseases, 10^th^ Revision. The registry was maintained by designated data managers who were blinded to the study analyses.

### Study population

We included patients (1) aged ≥ 18 years, (2) diagnosed with COVID-19 with a positive RT-PCR result for SARS-CoV-2 from an upper respiratory tract sample obtained by nasopharyngeal swab, and (3) admitted to the hospital for COVID-19 treatment because of oxygen requirement or risks for exacerbation. Patients who had been diagnosed with IBS before COVID-19 diagnosis were excluded.

### Data collection and definition

The collected data included patient demographics; comorbidities; date of a positive RT-PCR result for SARS-CoV-2; RT-PCR quantification cycle (Ct) for SARS-CoV-2; vital signs recorded by patient-monitoring devices and healthcare providers; laboratory data, such as white blood cell (WBC) count and hemoglobin, platelet, albumin, total bilirubin, aspartate aminotransferase, alanine transaminase, blood urea nitrogen, creatinine, and C-reactive protein (CRP) levels; medications for COVID-19, including dexamethasone, methylprednisolone, tocilizumab, and remdesivir; a descriptive record of GI symptoms; GI diseases diagnosed after admission; and the date and time for each collected data. Length of hospitalization, length of ICU admission, and survival status were also assessed.

Daily GI symptoms during hospital stay were recorded by registered nurses at least three times a day and included nausea, vomiting, diarrhea, abdominal distention, abdominal discomfort, and abdominal pain. The stool form was observed by a healthcare provider and classified using the Bristol Stool Scale. Diarrhea was defined as 6 or 7 on the Bristol scale [19], and constipation was defined as 1 or 2 on the Bristol scale or absence of defecation for 3 consecutive calendar days. IBS was clinically diagnosed by board certified physicians who completed gastroenterology training using the Rome IV criteria with at least 3 months of observation of symptoms [20, 21]. In addition, among patients with data available on the duration of all medications and the timing of diagnosis, IBS was objectively confirmed either by (1) the date of IBS diagnosis that was ≥ 4 months after COVID-19 admission (at least 1-month interval before the initial IBS symptoms) or by (2) ≥ 3 months of use of medication for IBS with at least a 1-month interval before the initial IBS symptoms (therefore, ≥ 4 months of observation). Subtypes of IBS were not recorded in the registry and the follow-up periods varied across patients from 1 month to 1 year, depending on the date of admission for COVID-19 and presence of symptoms.

### Outcome measures

The primary outcome was development of IBS after recovery from COVID-19. The secondary outcomes included in-hospital mortality and ICU-free and hospital-free days (a composite of in-hospital death and ICU/hospital length of stay, defined as the number of days alive and out of the ICU or hospital, respectively) up to day 30, in which the days were counted from the day of a positive RT-PCR result for SARS-CoV-2.

### Statistical analysis

Patient data were classified into IBS and non-IBS groups based on the diagnosis of IBS following COVID-19. To identify possible risks for development of IBS following COVID-19, univariate analyses were performed on patient background characteristics, including demographics, vital signs, and laboratory findings on hospital admission, treatment for COVID-19, and severity of COVID-19 (requirement of ICU admission). The secondary outcomes were also compared between patients with and without IBS to further capture the clinical features of patients who developed IBS, although they were not considered risk factors for IBS.

Details of GI symptoms, including the type of GI symptoms, days from COVID-19 diagnosis to symptoms, and laboratories at the onset of any initial GI symptoms, were compared between the groups using univariate analyses. Then, adjusted analyses were performed using multivariate logistic models with the backward stepwise method to validate the potential risk of development of IBS. Covariates for the adjusted analysis were selected from the patient background characteristics and GI symptom-related data, considering the results of the univariate analyses. The number of covariates was determined following standard methods of sample size estimation for multivariate logistic regression: one to two potential predictors for ten outcome events [22].

Considering that the number of included patients was expected to be low because of the single-center design, a sensitivity analysis was conducted using bootstrapping (resampling the model 1,000 times) [23], and multivariate logistic regression analysis was repeated with the same covariates for the adjusted analysis. Additionally, the association between each potential trigger for IBS and the severity of COVID-19 was examined by subgroup analyses. Each potential risk factor identified in the adjusted analyses was compared between patients with and without IBS in subgroups determined by the requirement for ICU admission (severe vs. mild-to-moderate COVID-19).

Descriptive statistics are presented as median (interquartile range) or number (percentage). Patient characteristics and GI symptom-related data were compared using the Mann–Whitney U test, chi-square test, or Fisher’s exact test, as appropriate. In hypothesis testing, a two-sided α threshold of 0.05 was considered significant. To avoid over- or under-estimating potentially important differences by a limited sample size, results are described using standardized differences and 95% confidence intervals (CI), wherein a standardized difference > 0.3 was considered a non-negligible difference. All statistical analyses were conducted using IBM SPSS Statistics for Windows (version 27.0; IBM Corp., Armonk, NY, USA) and Microsoft Excel (Microsoft Corp., Redmond, WA, USA).

## Results

### Patient characteristics

**Fig. 1:**
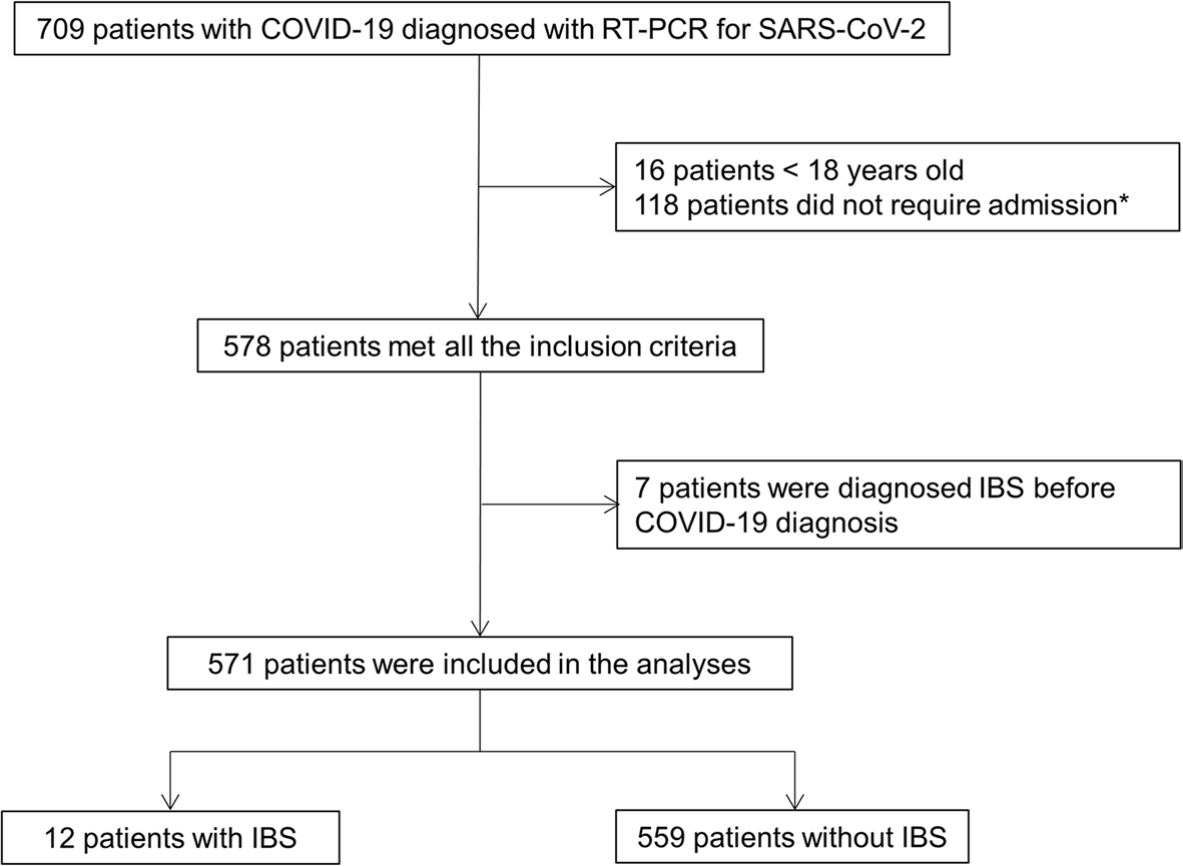
Patient flow diagram. Among 709 patients with COVID-19 during the study period, 578 were admitted to the study institution and met all inclusion criteria. Seven patients were diagnosed with IBS before COVID-19 diagnosis and excluded; therefore, 571 patients were eligible for this study. Among them, 12 (2.1%) patients were diagnosed with IBS after COVID-19. *This included 3 patients aged < 18 years. COVID-19, coronavirus disease 2019; IBS, irritable bowel syndrome

Among the 709 patients diagnosed with COVID-19 during the study period, 578 were admitted to the study institution and met all inclusion criteria. Seven patients were diagnosed with IBS before COVID-19 diagnosis; therefore, 571 patients were eligible for this study. Among them, 12 (2.1%) patients were diagnosed with IBS following recovery from COVID-19. In patients with objectively confirmed IBS, the median duration from admission for COVID-19 to IBS diagnosis was 5 months and the median duration of medication use for IBS was 4 months. A patient flow diagram is presented in Fig. 1.

The patients’ background characteristics are summarized in Table 1. Patients diagnosed with IBS had higher WBC counts upon hospital admission, and more patients with IBS had severe COVID-19 (required ICU admission) than those without IBS (6.1 [5.5–8.1] vs. 4.8 [3.9–6.2] 10^3^/μL, *p* = 0.010 and 4 [33.3%] vs. 63 [11.3%], *p* = 0.041, respectively). Moreover, non-negligible differences (standardized difference > 0.3) were identified in the treatment for COVID-19, with less frequent use of dexamethasone and tocilizumab among patients with IBS than in those without IBS. The possible subtypes of IBS determined by medications are summarized in Table 2. In addition, the characteristics of patients with objectively confirmed IBS is shown in Table S1, which is similar to those of 12 patients with clinically diagnosed IBS.

**Table 1:** Characteristics of patients with COVID-19

	IBS		No IBS		*p* value	Standardized Difference
Case	12		559			
Age, years, median (IQR)	54	(36–64)	50	(34–61)	0.773	0.061
Sex, male, n (%)	8	(66.7%)	368	(65.8%)	1.000	0.018
BMI, median (IQR)	22	(17–27)	23	(21–27)	0.405	0.167
Ct value on RCP for SARS-CoV-2^a^, median (IQR)	30	(21–34)	26	(20–32)	0.735	0.064
Comorbidity, n (%)						
Hypertension	2	(16.7%)	61	(10.9%)	0.631	0.167
Diabetes mellitus	0	(0.0%)	39	(7.0%)	1.000	0.387
Heart failure	0	(0.0%)	4	(0.7%)	1.000	0.120
Ischemic heart disease	0	(0.0%)	17	(3.0%)	1.000	0.250
Asthma	1	(8.3%)	34	(6.1%)	0.535	0.087
COPD	0	(0.0%)	6	(1.1%)	1.000	0.147
Interstitial pneumoniae	0	(0.0%)	3	(0.5%)	1.000	0.104
CKD	1	(8.3%)	7	(1.3%)	0.157	0.336
Cirrhosis	0	(0.0%)	2	(0.4%)	1.000	0.085
Vital signs on admission, median (IQR)						
Respiratory rate, /min	17	(12–21)	17	(15–20)	0.482	0.340
Heart rate, /min	70	(49–95)	69	(60–81)	0.664	0.043
Systolic blood pressure, mmHg	111	(105–118)	116	(105–127)	0.307	0.353
Body temperature, ℃	36.7	(36.3–37.9	36.6	(36.3–37.1)	0.890	0.250
Laboratory on admission, median (IQR)						
WBC, 10^3^/μL	6.1	(5.5–8.1)	4.8	(3.9–6.2)	**0.010**	0.612
WBC fractions, %						
- Banded neutrophil	1	(1–2)	3	(1–6)	0.131	1.041
- Segmented neutrophil	90	(79–91)	65	(54–76)	0.050	1.300
- Lymphocyte	19	(8–29)	23	(16–31)	0.155	0.486
- Monocyte	5	(4–8)	7	(5–10)	0.080	0.478
Hgb, g/dL	13.6	(12.1–15.6)	14.4	(13.1–15.6)	0.574	0.094
Platelet, 10^3^/μL	210	(170–270)	190	(160–240)	0.518	0.154
Albumin, g/dL	3.8	(3.2–4.2)	3.9	(3.5–4.2)	0.383	0.291
Total bilirubin, mg/dL	0.6	(0.4–1.0)	0.6	(0.5–0.8)	0.983	0.014
AST, IU/L	34	(19–56)	29	(21–43)	0.639	0.108
ALT, IU/L	19	(14–45)	26	(16–43)	0.654	0.098
BUN, mg/dL	14	(10–16)	12	(10–16)	0.437	0.191
Creatinine, mg/dL	0.8	(0.6–1.1)	0.8	(0.7–1.0)	0.980	0.037
CRP, mg/dL	2.8	(0.4–12.3)	1.8	(0.3–5.3)	0.431	0.406
Treatment, n (%)						
Dexamethasone	2	(16.7%)	183	(32.7%)	0.354	0.379
Methylprednisolone	2	(16.7%)	18	(3.2%)	0.062	0.461
Tocilizumab	0	(0.0%)	55	(9.8%)	0.617	0.467
Remdesivir	4	(33.3%)	247	(44.2%)	0.564	0.224
ICU admission, n (%)	4	(33.3%)	63	(11.3%)	**0.041**	0.550

*COVID-19* Novel coronavirus disease 2019, *IBS* Irritable bowel syndrome, *IQR* Interquartile range, *BMI* Body mass index, *Ct* Cycle of quantification, *PCR* Polymerase chain reaction, *SARS-CoV-2* Severe acute respiratory syndrome coronavirus 2, *COPD* Chronic obstructive pulmonary disease, *CKD* Chronic kidney disease, *WBC* White blood cell count, *Hgb* Hemoglobin, *AST* Aspartate aminotransferase, *ALT* Alanine transaminase, *BUN* Blood urea nitrogen, *CRP* C-reactive protein, *ICU* Intensive care unit

^a^When multiple samples were obtained at the same time, Ct values were averaged

**Table 2:** Medications and possible subtypes of IBS

	n (%)
Medications	
Anti-diarrheal agents	4 (33.3%)
Probiotics/laxatives	10 (83.3%)
Possible subtypes^a^	
IBS with diarrhea (IBS-D)	0 (0.0%)
IBS with constipation (IBS-C)	3 (25.0%)
Mixed IBS (IBS-M)	7 (58.3%)

*IBS* Irritable bowel syndrome

^a^Subtypes of IBS were determined by medications for IBS. Subtypes could not be determined in two patients due to unavailable data on medications

Regarding secondary outcomes, patients with IBS had fewer ICU-free days compared to those without IBS, while in-hospital mortality and hospital-free days were comparable between patients with and without IBS (Table S2).

### GI symptoms and risk for development of IBS

GI symptoms and related information are summarized in Table 3. Nausea and diarrhea were more frequently identified in patients with IBS than in those without (3 [25.0%] vs. 32 [5.7%], *p* = 0.032 and 10 [83.3%] vs. 245 [43.8%], *p* = 0.008, respectively). Meanwhile, other GI symptoms, including abdominal pain, number of days from COVID-19 diagnosis (positive PCR) to onset of symptoms, and laboratory parameters at the onset of initial GI symptoms were comparable between the groups; however, the number of days from COVID-19 diagnosis to onset of nausea was relatively longer in patients with IBS (10 [8-16] vs. 3 [1-10] days, standardized difference = 0.724). Moreover, the WBC count and CRP levels at the onset of initial GI symptoms were relatively higher in patients with IBS than in those without IBS.

**Table 3:** Abdominal symptoms in patients with COVID-19

	IBS	No IBS	*p* value	Standardized Difference
Case	12	559		
Abdominal symptoms, n (%)				
Nausea	3 (25.0%)	32 (5.7%)	**0.032**	0.555
Vomiting	0 (0.0%)	17 (3.0%)	1.000	0.251
Diarrhea	10 (83.3%)	245 (43.8%)	**0.008**	0.900
Constipation	5 (41.7%)	241 (43.1%)	1.000	0.029
Abdominal pain	0 (0.0%)	5 (0.9%)	1.000	0.134
Abdominal distention	0 (0.0%)	9 (1.6%)	1.000	0.181
Days to symptoms from PCR, median (IQR)				
Nausea	10 (8–16)	3 (1–10)	0.096	0.724
Vomiting	-	5 (1–10)		
Diarrhea	1 (0–6)	2 (1–4)	0.807	0.000
Constipation	3 (2–12)	3 (2–5)	0.972	0.262
Abdominal pain	-	2 (0–7)		
Abdominal distention	-	10 (7–22)		
Laboratory at initial abdominal symptom, median (IQR)				
WBC, 10^3^/μL	6.3 (4.3–9.1)	5.1 (3.9–6.9)	0.186	0.448
Hgb, g/dL	13.4 (11.7–14.9)	13.7 (12.3–14.9)	0.705	0.019
Platelet, 10^3^/μL	210 (170–260)	200 (160–270)	0.985	0.106
Albumin, g/dL	3.0 (2.6–3.9)	3.4 (2.9–3.7)	0.344	0.340
Total bilirubin, mg/dL	0.4 (0.4–0.7)	0.6 (0.4–0.7)	0.447	0.215
AST, IU/L	25 (17–33)	29 (22–42)	0.134	0.320
ALT, IU/L	23 (12–72)	27 (17–46)	0.617	0.058
BUN, mg/dL	17 (9–30)	14 (10–20)	0.362	0.283
Creatinine, mg/dL	0.8 (0.6–1.2)	0.8 (0.6–1.0)	0.799	0.020
CRP, mg/dL	4.3 (1.2–11.1)	2.2 (0.6–5.2)	0.156	0.369

*COVID-19* Novel coronavirus disease 2019, *IBS* Irritable bowel syndrome, *PCR* Polymerase chain reaction, *IQR* Interquartile range, *WBC* White blood cell count, *Hgb* Hemoglobin, *AST* Aspartate aminotransferase, *ALT* Alanine transaminase, *BUN* Blood urea nitrogen, *CRP* C-reactive protein

Adjusted analysis was conducted using the multivariate logistic regression model to identify the risk factors for development of IBS following COVID-19; WBC count on admission, requirement of ICU admission (severe COVID-19), nausea, and diarrhea were entered. As the number of covariates in the logistic model needed to be limited because of the small sample size, they were selected using the backward stepwise method. Nausea and diarrhea were selected in the final model and determined as risk factors for the development of IBS (odds ratio [OR], 4.00; 95% CI, 1.01–15.84; *p* = 0.049 and OR, 5.64; 95% CI, 1.21–26.31; *p* = 0.028, respectively; Table 4). Sensitivity analysis with bootstrapping similarly revealed an association between diarrhea and development of IBS following COVID-19, but not for the relationship between nausea and consecutive IBS diagnoses (Table 4).

**Table 4:** Abdominal symptoms and IBS in multivariate analysis

	OR (95% CI)		*p* value
Multivariate analysis^a^			
Nausea	4.00	(1.01–15.84)	0.049
Diarrhea	5.64	(1.21–26.31)	0.028
Sensitivity analysis with bootstrapping^b^			
Nausea	3.82	(0.00–12.27)	
Diarrhea	4.83	(1.17–)^c^	

*IBS* Irritable bowel syndrome, *OR* Odds ratio

^a^Multivariate logistic regression with the backward stepwise method was performed using white blood cell count on admission, ICU admission, nausea, and diarrhea

^b^Bootstrap analysis was conducted with resampling the model 1000 times

^c^Upper limit of 95% CI was not shown due to too large number

In the subgroup analyses (Table S3), nausea was associated with the development of IBS in patients with severe COVID-19 (3 [75.0%] vs. 10 [15.9%], *p* = 0.021) but not in those with mild-to-moderate COVID-19. Conversely, the association between diarrhea and the development of IBS following COVID-19 was not significant in patients with severe or non-severe COVID-19.

### Daily GI symptoms in patients with IBS

**Fig. 2:**
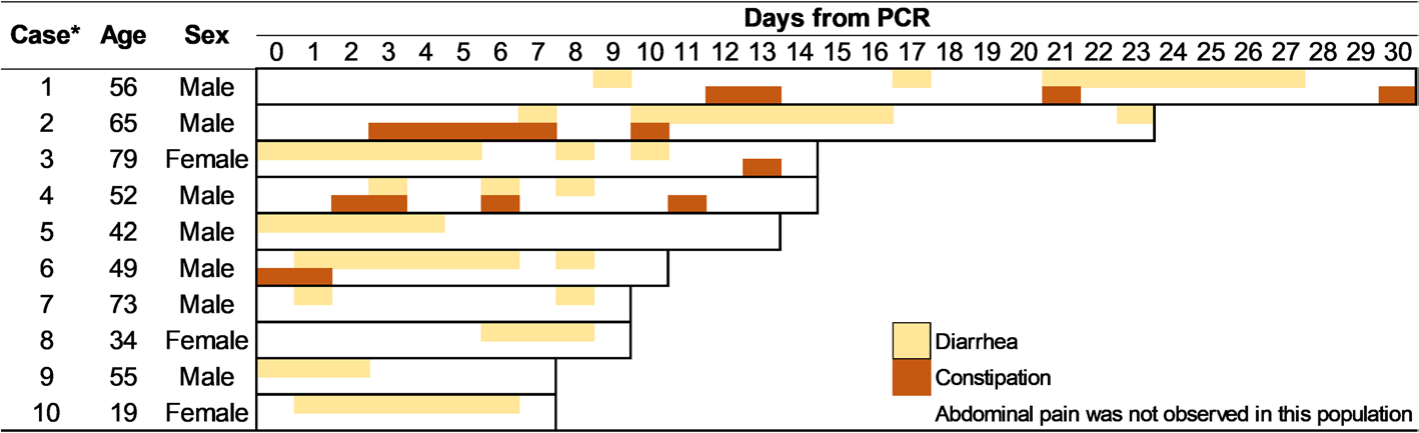
Gastrointestinal symptoms in patients who developed irritable bowel syndrome following COVID-19. Diarrhea and constipation (including abdominal distention) are summarized in each patient who developed IBS following COVID-19 as typical symptoms of IBS. Half of the patients with IBS had both diarrhea and constipation until discharge, and constipation was frequently followed by diarrhea. Abdominal pain was not observed in this population. ^*^Among the 12 patients who were diagnosed with IBS following COVID-19, the information of two patients who had missing data on daily gastrointestinal symptoms throughout the hospital stay is not presented in the figure. PCR, polymerase chain reaction; COVID-19, coronavirus disease 2019; IBS, irritable bowel syndrome

Among the 12 patients diagnosed with IBS following COVID-19, 10 had no missing data on daily GI symptoms throughout the hospital stay. Diarrhea and constipation (including abdominal distention) were then summarized as typical symptoms of IBS (Fig. 2), whereas abdominal pain was not observed in any patient diagnosed with IBS following COVID-19.

Half of the patients with IBS had both diarrhea and constipation until discharge, and constipation was frequently followed by diarrhea. While many patients had diarrhea for at least 5 days, almost all of them had diarrhea or constipation for more than half of the length of their hospitalization.

## Discussion

In this retrospective study, IBS was diagnosed in approximately 2% of patients with COVID-19, and analyses utilizing detailed daily GI symptoms revealed that diarrhea and nausea during COVID-19 treatment were significantly associated with the development of IBS. While several clinical features were identified as potential risk factors for development of IBS due to SARS-CoV-2 infection, the multivariate adjusted analysis revealed that diarrhea and nausea could precede early signs of post-infectious IBS following COVID-19.

Several pathophysiological mechanisms underlying the association between diarrhea/nausea and the development of IBS following COVID-19 should be considered. First, direct infection with SARS-CoV-2 in the GI tract would cause post-infectious IBS [24–26]. As medications for IBS were initiated at 1 month after COVID-19 diagnosis in this study, inflammation of enterocytes would take 1 month to develop post-infectious IBS. Second, given that ACE-2-mediated function is known to abnormally increase mucosal permeability and GI motility [27, 28], SARS-CoV-2 would have bound to the ACE-2 receptor in the GI tract among patients who developed IBS in this study. Notably, while previous studies have suggested that GI symptoms could persist after recovery from COVID-19 until the development of post-infectious IBS [29, 30], the present study indicated a latent period (1 month) until initiation of IBS medications. Third, the background characteristics of patients would affect the gut microbiota, which predisposes patients to the development of IBS [9, 16]. As ICU admission was more frequent in patients who had post-infectious IBS in this study, nausea and diarrhea could have been manifested as adverse effects of various medications, including vasopressors and sedatives, in such a population. Interestingly, Clostridioides difficile infection and antibiotic-related diarrhea were not identified in any patient with IBS in this study.

Although the severity of COVID-19 was not validated as an independent predictor of IBS after SARS-CoV-2 infection in this study, possible triggers for IBS would exist in patients with severe COVID-19. Cytokine imbalance frequently occurs in cases of severe COVID-19 [31], and gut functions are disturbed through destructive autoinflammation [21], which can eventually induce IBS. Moreover, psychological stress in severe COVID-19 can interfere with the brain-gut axis [32]. Here, higher WBC counts with a relatively higher rate of segmented neutrophils on hospital admission, relatively higher WBC counts and CRP levels at the onset of GI symptoms, and prolonged ICU stays were observed among patients with IBS following COVID-19. The relationship between the severity of COVID-19 and the development of IBS should be further examined in future studies with larger sample sizes.

Detailed GI symptoms during the treatment of COVID-19 were extensively examined in this study, and some distinctive daily changes in GI symptoms would be considered as early clinical signs of IBS following COVID-19. Although the GI symptoms during hospitalization for COVID-19 treatment are not necessarily equal to those for the diagnosis of IBS, predominant stool patterns until discharge were similar to those of IBS with diarrhea, known as IBS-D, or mixed IBS, known as IBS-M [33]. Conversely, abdominal pain during COVID-19 treatment was not necessary for the development of post-infectious IBS.

The present study obtained data regarding daily GI symptoms from real-world practice that were retrieved directly from a hospital information system. Therefore, a more robust association between GI symptoms during hospitalization for the treatment of COVID-19 and the development of IBS would be expected in this study compared with previous studies.

This study has some limitations. First, we utilized data from the Donner Registry to retrieve information directly from the hospital information system, which does not record the details of IBS diagnoses. Therefore, our results may differ if the GI symptoms for IBS diagnosis were over- or underestimated, although the Rome IV criteria were referred to in diagnosing IBS in this study. However, it should be noted that patients with objectively confirmed IBS, based on the details of medications and the timing of diagnosis, had similar characteristics to those with clinically diagnosed IBS. Second, the study was conducted at a single center with limited sample size and follow-up period. Therefore, other potential risks, such as the severity of COVID-19 and psychiatric status should be further examined in future studies. In addition, considering that new GI symptoms could emerge later than 3 months after recovery from COVID-19 [10], the prevalence of post-infectious IBS should be further examined in longer follow-up periods. Third, during the COVID-19 pandemic, several novel medications have been developed and reported to improve outcomes [3–5]. Therefore, some medications can mitigate the degree of viral load and may reduce the risk of the development of IBS. Furthermore, patients with a previous diagnosis of IBS were excluded in this study. As the accurate diagnosis of IBS is difficult for non-gastroenterologist, these patients may have been inappropriately excluded. Finally, as we investigated only patients who required hospitalization, our results cannot be generalized to patients with mild or no COVID-19 symptoms.

## Conclusions

Diarrhea and nausea during hospitalization for COVID-19 treatment were associated with the development of IBS after SARS-CoV-2 infection, while IBS was rarely diagnosed (2%) in patients with COVID-19. The severity of COVID-19 and IBS subtypes following COVID-19 should be examined further in future studies.

## Supplementary Information

**Additional file1:** **Table S1.** Characteristics of patients with objectively confirmed irritable bowel syndrome. **Table S2.** Secondary outcomes in patients with and without IBS. **Table S3.** Abdominal symptoms and IBS in subgroups.

## Data Availability

The data for this study are available from the Donner Registry of the Keio Donner Project; however, restrictions apply to the availability of these data, which were used under a license for the present study, so they are not publicly available. However, data are available from the corresponding author upon reasonable request and with permission of Keio Donner Project at Keio University School of Medicine for researchers who meet the criteria for accessing confidential data.

## Abbreviations

COVID-19: Coronavirus disease 2019
GI: Gastrointestinal
ACE-2: Angiotensin-converting enzyme-2
SARS-CoV-2: Severe acute respiratory syndrome coronavirus-2
FGID: Functional GI disorders
IBS: Irritable bowel syndrome
RT-PCR: Reverse transcription polymerase chain reaction
MV: Mechanical ventilation
ICU: Intensive care unit
WBC: White blood cell
CRP: C-reactive protein
CI: Confidence intervals
OR: Odds ratio
