# Supplementary material for: Early symptoms preceding post-infectious irritable bowel syndrome following COVID-19: a retrospective observational study incorporating daily gastrointestinal symptoms
Source: BMC Gastroenterol. 2023 Apr 5;23:108. doi: 10.1186/s12876-023-02746-y (PMC10075174; doi:10.1186/s12876-023-02746-y)
Supplement: Supplementary file 1 — Additional file1: Table S1. Characteristics of patients with objectively confirmed irritable bowel syndrome. Table S2. Secondary outcomes in patients with and without IBS. Table S3. Abdominal symptoms and IBS in subgroups. [file 12876_2023_2746_MOESM1_ESM.docx]

| Table S1. Characteristics of patients with objectively confirmed irritable bowel syndrome | | | | | | |  |
| --- | --- | --- | --- | --- | --- | --- | --- |
|  |  | IBS | | No IBS | | p value |  |
| Case | | 6 | | 559 | |  |  |
| Age, years, median (IQR) | | 45 | (22-67) | 50 | (34-61) | 0.590 |  |
| Sex, male, n (%) | | 3 | (50.0%) | 368 | (65.8%) | 0.418 |  |
| BMI, median (IQR) | | 21 | (16-26) | 23 | (21-27) | 0.188 |  |
| Ct value on RCP for SARS-CoV-2*, median (IQR) | | 27 | (14-33) | 26 | (20-32) | 0.725 |  |
| Comorbidity, n (%) | |  |  |  |  |  |  |
|  | Hypertension | 2 | (33.3%) | 61 | (10.9%) | 0.137 |  |
|  | Diabetes mellitus | 0 | (0.0%) | 39 | (7.0%) | 1.000 |  |
|  | Heart failure | 0 | (0.0%) | 4 | (0.7%) | 1.000 |  |
|  | Ischemic heart disease | 0 | (0.0%) | 17 | (3.0%) | 1.000 |  |
|  | Asthma | 1 | (16.7%) | 34 | (6.1%) | 0.320 |  |
|  | COPD | 0 | (0.0%) | 6 | (1.1%) | 1.000 |  |
|  | Interstitial pneumoniae | 0 | (0.0%) | 3 | (0.5%) | 1.000 |  |
|  | CKD | 1 | (16.7%) | 7 | (1.3%) | 0.082 |  |
|  | Cirrhosis | 0 | (0.0%) | 2 | (0.4%) | 1.000 |  |
| Vital signs on admission, median (IQR) | |  |  |  |  |  |  |
|  | Respiratory rate, /min | 17 | (15-20) | 17 | (15-20) | 0.102 |  |
|  | Heart rate, /min | 74 | (55-86) | 69 | (60-81) | 0.914 |  |
|  | Systolic blood pressure, mmHg | 112 | (101-125) | 116 | (105-127) | 0.598 |  |
|  | Body temperature, ℃ | 36.3 | (36.2-37.5) | 36.6 | (36.3-37.1) | 0.510 |  |
| Laboratory on admission, median (IQR) | |  |  |  |  |  |  |
|  | WBC, 10^3^/μL | 6.8 | (5.7-8.7) | 4.8 | (3.9-6.2) | 0.013 |  |
|  | WBC fractions, % |  |  |  |  |  |  |
|  | - Banded neutrophil | - |  | 3 | (1-6) | - |  |
|  | - Segmented neutrophil | - |  | 65 | (54-76) | - |  |
|  | - Lymphocyte | 21 | (7-32) | 23 | (16-31) | 0.486 |  |
|  | - Monocyte | 6 | (3-10) | 7 | (5-10) | 0.482 |  |
|  | Hgb, g/dL | 13.3 | (11.8-14.4) | 14.4 | (13.1-15.6) | 0.118 |  |
|  | Platelet, 10^3^/μL | 230 | (170-290) | 190 | (160-240) | 0.248 |  |
|  | Albumin, g/dL | 3.8 | (2.9-4.4) | 3.9 | (3.5-4.2) | 0.847 |  |
|  | Total bilirubin, mg/dL | 0.4 | (0.4-1.0) | 0.6 | (0.5-0.8) | 0.171 |  |
|  | AST, IU/L | 20 | (18-35) | 29 | (21-43) | 0.119 |  |
|  | ALT, IU/L | 15 | (13-25) | 26 | (16-43) | 0.086 |  |
|  | BUN, mg/dL | 14 | (10-35) | 12 | (10-16) | 0.288 |  |
|  | Creatinine, mg/dL | 0.9 | (0.5-1.5) | 0.8 | (0.7-1.0) | 0.838 |  |
|  | CRP, mg/dL | 0.9 | (0.1-7.7) | 1.8 | (0.3-5.3) | 0.725 |  |
| Treatment, n (%) | |  |  |  |  |  |  |
|  | Dexamethasone | 0 | (0.0%) | 183 | (32.7%) | 0.184 |  |
|  | Methylprednisolone | 2 | (33.3%) | 18 | (3.2%) | 0.016 |  |
|  | Tocilizumab | 0 | (0.0%) | 55 | (9.8%) | 1.000 |  |
|  | Remdesivir | 1 | (16.7%) | 247 | (44.2%) | 0.237 |  |
| ICU admission, n (%) | | 2 | (33.3%) | 63 | (11.3%) | 0.144 |  |
| COVID-19 = Novel coronavirus disease 2019, IBS = irritable bowel syndrome, IQR = interquartile range, BMI = body mass index, Ct = cycle of quantification, PCR = polymerase chain reaction, SARS-CoV-2 = severe acute respiratory syndrome coronavirus 2, COPD = chronic obstructive pulmonary disease, CKD = chronic kidney disease, WBC = white blood cell count, Hgb = hemoglobin, AST = aspartate aminotransferase, ALT = alanine transaminase, BUN = blood urea nitrogen, CRP = C-reactive protein, and ICU = intensive care unit. *When multiple samples were obtained at the same time, Ct values were averaged. | | | | | | |  |
|  |  |  |  |  |  |  |  |
|  |  |  |  |  |  |  |  |
|  |  |  |  |  |  |  |  |
|  |  |  |  |  |  |  |  |
|  |  |  |  |  |  |  |  |

| Table S2. Secondary outcomes in patients with and without IBS | | | |  |
| --- | --- | --- | --- | --- |
|  | IBS | No IBS | p value |  |
| In-hospital mortality, *n (%)* | 0 (0.0%) | 15 (2.7%) | 1.000 |  |
| ICU-free days to day 30, *days, median (IQR)* | 30 (23–30) | 30 (30–30) | 0.013 |  |
| Hospital-free days to day 30, *days, median (IQR)* | 19 (9–23) | 21 (17–24) | 0.197 |  |
| IBS = irritable bowel syndrome, ICU = intensive care unit, and IQR = interquartile range. | | | |  |
|  |  |  |  |  |

| Table S3. Abdominal symptoms and IBS in subgroups | | | | |  |
| --- | --- | --- | --- | --- | --- |
|  |  | IBS | No IBS | p value |  |
| Nausea | |  |  |  |  |
|  | Severe COVID-19* | 3/4 (75.0%) | 10/63 (15.9%) | 0.021 |  |
|  | Mild to moderate COVID-19 | 0/8 (0.0%) | 22/496 (4.4%) | 1.000 |  |
| Diarrhea | |  |  |  |  |
|  | Severe COVID-19* | 4/4 (100.0%) | 54/63 (85.7%) | 1.000 |  |
|  | Mild to moderate COVID-19 | 6/8 (75.0%) | 191/496 (38.5%) | 0.061 |  |
| IBS = irritable bowel syndrome and COVID-19 = Novel coronavirus disease 2019. *Severe COVID-19 was defined as those who required ICU admission. | | | | |  |
|  |  |  |  |  |  |
